# Music, body, and machine: gesture-based synchronization in human-robot musical interaction

**DOI:** 10.3389/frobt.2024.1461615

**Published:** 2024-12-05

**Authors:** Xuedan Gao, Amit Rogel, Raghavasimhan Sankaranarayanan, Brody Dowling, Gil Weinberg

**Affiliations:** Robotic Musicianship Lab, Center for Music Technology, Georgia Institute of Technology, Atlanta, GA, United States

**Keywords:** human-robot interaction, synchronization, robotic gestures, robotic musicianship, robots, music

## Abstract

Musical performance relies on nonverbal cues for conveying information among musicians. Human musicians use bodily gestures to communicate their interpretation and intentions to their collaborators, from mood and expression to anticipatory cues regarding structure and tempo. Robotic Musicians can use their physical bodies in a similar way when interacting with fellow musicians. The paper presents a new theoretical framework to classify musical gestures and a study evaluating the effect of robotic gestures on synchronization between human musicians and Shimon - a robotic marimba player developed at Georgia Tech. Shimon utilizes head and arm movements to signify musical information such as expected notes, tempo, and beat. The study, in which piano players were asked to play along with Shimon, assessed the effectiveness of these gestures on human-robot synchronization. Subjects were evaluated for their ability to synchronize with unknown tempo changes as communicated by Shimon’s ancillary and social gestures. The results demonstrate the significant contribution of non-instrumental gestures to human-robot synchronization, highlighting the importance of non-music-making gestures for anticipation and coordination in human-robot musical collaboration. Subjects also indicated more positive feelings when interacting with the robot’s ancillary and social gestures, indicating the role of these gestures in supporting engaging and enjoyable musical experiences.

## 1 Introduction

Music and movement have co-evolved in many cultures, serving important functions in our social behavior (Cross, 2001). One manifestation of the connection between music and movement can be seen when musicians make complementary, sometimes subconscious, gestures while playing. These non-music-making accompanying movements, also known as ancillary gestures (Wanderley, 1999), provide valuable insights into a musician’s expression and intent. When musicians play together, they frequently use ancillary and social gestures to communicate information about their mood, timing, and personal interpretation of the music, which can be helpful in assisting collaborators in adjusting their own musical performance.

For musical robots, however, designers often focus on optimizing performance by minimizing unnecessary movement of actuators, as can be demonstrated by examples such as the Waseda robotic flute player (Solis et al., 2006), Singer’s Guitar Bot (Singer et al., 2003), and the Georgia Tech’s anthropomorphic percussionist Haile (Weinberg and Driscoll, 2006). This efficiency-driven approach cannot convey rich expressive information to the robot’s musical collaborators. We believe that a gesture-aware approach can be an effective tool for maintaining expressive human-robot connection. In particular, ancillary and social gestures can help musicians anticipate, coordinate and synchronize their actions with a robotic musician.

The study presented in this paper investigates the effectiveness of non-instrumental gestures for human-robot musical synchronization. We use Shimon (Hoffman and Weinberg, 2010b), a marimba-playing robot developed by Georgia Tech’s Robotic Musicianship Group, as a platform for the study. Since Shimon’s head is not used for generating sound, its gestures can be dedicated to social cues that can communicate current and future tempo interpretation to his collaborators. By assessing human pianists’ ability to synchronize with these tempo changes while the robot plays the accompaniment, we aim to understand the effectiveness of these gestures in improving human-robot synchronization and engagement.

## 2 Related work

To develop gestures that can help musicians synchronize with Shimon, we examine research on visual cues in human-robot interaction outside of music as well as visual communication between human musicians.

### 2.1 Embodiment in musical performances

Different types of musical communication rely on direct gestures, where an exchange of sensory information, facilitated by physical movements, allows for expressive interactions (Leman, 2016). Jeanne and Jacob found that people understand others’ motor intentions by mimicking each other’s movements (Jacob and Jeannerod, 2005; Jeannerod, 2003). Our human ability to naturally create these gestures is central to the concept of embodied communication in music (Bishop and Goebl, 2018).

Cadoz and Wanderly categorized performer gestures into instrumental gestures and ancillary gestures (Cadoz, 1988). According to their definition, instrumental gestures (also referred to as effective gestures by Delalande (Delalande, 1988)) directly cause the excitation or modification of the instrument. Ancillary gestures (Wanderley and Depalle, 2004), also referred to as ‘non-obvious’ (Wanderley, 1999) or ‘accompanist gestures’ (Cadoz and Wanderley, 2000), are accompanying body movements and postures not directly involved in sound production, but often convey artistic intention. In music group playing or in front of the audience, musicians also use communicative gestures which are primarily aimed at conveying information to other performers or observers (Jensenius and Wanderley, 2010).

Visual cues are useful for time coordination among musicians who use them to improve synchronization and enhance the overall expressiveness of the performance. Wanderley et al. examined the ancillary gestures of clarinetists during performances, focusing on their timing, relation to the score, different movement styles, and audience perception (Wanderley et al., 2005). Coorevits et al. suggested that expressive gestures can improve synchronization and tempo stability (Coorevits et al., 2020). Santos et al. conducted experiments exploring the reciprocity between ancillary gestures and music structure performed by expert musicians (Santos, 2019). Bishop et al. focused on specific ancillary gestures, highlighting the importance of musicians’ head gestures in signaling the onset of a piece and in coordinating time changes (Bishop and Goebl, 2018).

A few studies have investigated the synchronization mechanisms in music playing. Konvalinka et al. showed that mutual adaptation, not a leader-follower dynamic, is key to successful coordination in joint musical activities (Konvalinka et al., 2010). Walton et al. studied how musical context shapes coordination in improvisation, revealing patterns of collaboration through movement and playing behavior (Walton et al., 2018). Badino et al. showed that effective leadership in group coordination relies on shared information rather than unidirectional control (Badino et al., 2014). This study highlights the importance of synchronization in human-to-human interaction and the need for conducting similar studies in human-robot synchronization, as presented here.

### 2.2 Visual cues in human-robot interaction

Nonverbal cues play a crucial role in interpersonal communication, as a significant part of human interaction occurs on a nonverbal level (Andersen, 2014). Similarly, these nonverbal signals, such as gestures, facial expressions, and eye contact, are important for human-robot interaction. Urakami et al. suggested a framework integrating nonverbal cues based on human sensory systems into robot designs, showing that such cues enhance the liveliness and social engagement with robots (Urakami and Seaborn, 2023). Other research evaluated the importance of visual cues for coordinating actions and improving task efficiency in human-robot interactions (Breazeal et al., 2005; Ganesan et al., 2018; Hoffman and Weinberg, 2010c). Obo et al. showed the helpful role of visual cues in indicating turn-taking between a human and a robot (Obo and Takizawa, 2022). Body language has also been shown to be an effective tool for robots to convey emotions. Beck et al. developed a system for humanoid robots that allows them to express emotions through body language, enhancing their perceived expressiveness and naturalness, synchronization, and fluency (Beck et al., 2012). These results are also supported by our previous findings, where we created a framework for HRI synchronization (Weinberg et al., 2006) and emotion-driven robotic gestures (Savery et al., 2024).

### 2.3 Robotic musicianship

Robotic musicianship is defined as the integration of the physical creation of music through robotic means with the algorithmic processes that enable machines to reason about and engage in music-related activities (Weinberg et al., 2020; Bretan and Weinberg, 2016). Robotic musicians have been developed to play a variety of musical instruments over the years, including piano (Kato et al., 1987), percussion (Weinberg and Driscoll, 2006), violin (Sankaranarayanan and Weinberg, 2021), and wind instruments (Solis et al., 2006; Uchiyama et al., 2023). Some robotic musicians have been designed to understand and interact with human musicians. For example, Cosentino et al. introduce a system that enables a robot musician to interpret an orchestra conductor’s gestures, allowing it to adapt its performance dynamically and enhance live musical communication with human musicians (Cosentino et al., 2014). Zahray et al. developed a method to sonify robots based on their movements (Zahray et al., 2020). Wang et al. proposed a theoretical framework for human-robot cooperative piano playing, utilizing an RNN for predicting chord progressions based on human input and a behavior-adaptive controller for temporal synchronization, achieving effective collaboration and real-time accompaniment (Wang et al., 2024). Studies have shown that simulated emotions conveyed by robotic musicians’ gestures can be interpreted by humans. For example, Burger et al. developed a three-wheeled robot capable of expressing emotions (Burger and Bresin, 2010). Savery et al. introduced a model based on music-driven emotional prosody and gestures, successfully expressing a range of emotions through musical phrases (Savery et al., 2019). Addressing human-robot synchronization in music, Lim et al. introduced a method enabling a musical robot to synchronize with human players by combining visual and audio inputs for real-time beat tracking (Lim et al., 2010). They also developed a robot that uses visual cues for ensemble synchronization by detecting the gestures of a human musician in various sections of the piece, such as start cue, end cue, and beat cue (Lim et al., 2012). While these works have focused on enabling robots to synchronize with human players using visual and audio inputs, little attention has been given to how robotic gestures can enhance how humans synchronize with the robot. The closest work to our current project has been conducted by Hoffman and Weinberg, who showed how our marimba-playing robot Shimon uses its music-making gestures to help human piano players synchronize with unanticipated tempo changes (Hoffman and Weinberg, 2011). No work has been done to our knowledge on the effect of social and ancillary gestures on HRI synchronization, which is the core contribution of the work presented here.

## 3 Gesture classification framework

In an effort to create a theoretical framework for our research, we would like to offer a new approach to musical gesture classification. While previous research addressed the mechanics of musical gesture creation, for example, separating instrumental gestures to their component of excitation, modification and selection of sound (Cadoz and Wanderley, 2000), our framework addresses the intent and function of such gestures, with a special emphasis on group play scenarios. For example, while lifting one’s arm before hitting a drum may fall under the category of modification of instrumental gestures according to Cadoz (Cadoz and Wanderley, 2000), we believe that such gestures also carry ancillary and social functions. We aim to classify gestures from the perspective of semiotic functions, as such classification better reflects how humans interpret the intentions and function of the gestures. We therefore offer the following classification for the musical gestures used in our study.

• Instrumental Gestures – gestures that are directly related to the excitation of sound (i.e., – hitting a drum or plucking a string).

• Ancillary Gestures – Accompanying gestures that anticipate or follow Instrumental Gestures, and indirectly affect the quality of sound excitation (i.e., – lifting the arm higher before playing a piano key or filling the lungs with air before blowing a trumpet).

• Social Gestures – Gestures that do not affect sound excitation, neither directly nor indirectly, rather are aimed to project intention and expression to fellow musicians or audiences (i.e., - bobbing one’s head to help others synchronize to the beat, turning towards fellow musicians to signify turn-taking, or exaggerating dance moves to excite audiences).

These three categories of gestures often appear in hybrid modes, for example, Ancillary and Instrumental gestures almost always come together; Ancillary and Social gestures are often combined in group playing scenarios, leading to Instrumental gestures. For humans in group play, social gestures often function as Ancillary gestures as well, as they affect the full human body and therefore the sound that is generated. In robots, designers can use specific actuators that are more direct in function. As a result, different actions in playing music can be disjointed. Therefore, instrumental and social gestures can appear in isolation. When designing and programming robotic musicians, a conscious effort may need to be made for a robot to express Ancillary and Social gestures. Robots such as Shimon can be programmed to project these social cues in response to the music being played as described below.

## 4 Shimon-the robotic marimba player

Shimon is a robotic marimba player who can improvise while providing visual cues to his collaborators (Hoffman and Weinberg, 2010a). Originally, Shimon utilized a variety of rule-based approaches for machine improvisation based on music theory rules. In one of these applications, the robot was programmed to improvise over a harmonic chord progression based on rules derived from canonical jazz improvisation textbooks (Bretan, 2017). In the past few years, Shimon moved to utilize a variety of data-driven approaches, from Genetic Algorithm and Markov Chains to recurrent neural networks, recombinancy and grammars. In one of these projects, a unit selection and concatenation method has been implemented as a means of generating music using a procedure based on ranking (Bretan et al., 2016). A generative model that combined a deep structured semantic model (DSSM) with an LSTM that predicted the next unit was implemented. The model was evaluated using objective metrics including mean rank and accuracy and with a subjective listening test in which expert musicians were asked to rate the quality of the musical output.

In another project, we developed a ConvNet model that utilized a symmetrical encoder-decoder architecture (Savery and Weinberg, 2022). Here, the outputs of the encoding layers were appended to the inputs of corresponding decoding layers. This model was distinct from other ConvNet models as it returned an output of the same size as input, and essentially performed a classification on every value from the input. The data format for the transcribed improvisation was arranged in beats such that time steps relative to the beginning of each beat were stacked on top of one another. This allowed the model to learn relatively coherent musical structure and discover temporal dependencies within each phrase. We also used transfer-learning via the T5 transformer model to generate lyrics for Shimon (Ram et al., 2021), which were later used to fit melodic lines composed by a human for Shimon to sing (Savery et al., 2021). In its most recent project, Shimon used a real-time system to listen to a rapper and respond with its own verbal responses based on linguistic prompts from the human (Savery and Weinberg, 2020).

### 4.1 Mechanical design

Mechanically, Shimon is comprised of two systems: one for music generation using its arms through a combination of ancillary and instrumental gestures, and the other for social gestures using its head.

**FIGURE 1:**
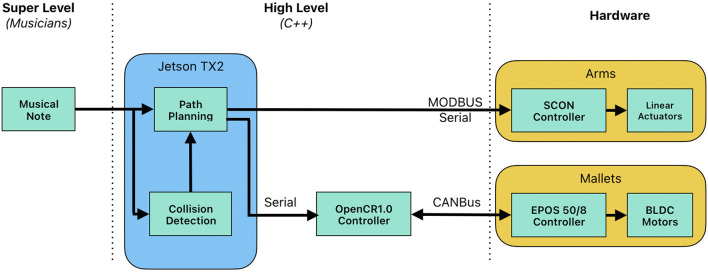
Shimon system flow chart.

The music-playing system features four sliding arms, each equipped with two striking mallets. The front mallets are designed to play sharps and flats on the marimba, while the back mallets handle natural notes. All four arms are mounted on a single IAI LSA-S linear slider, allowing coordinated movement of the arms along the marimba. To accommodate the increased complexity in Shimon’s playing. Shimon’s striking mechanism was recently upgraded from solenoids to Brushless DC motors (BLDC), which allows for a wider dynamic range, faster speeds, and more musical expression (Yang et al., 2020). We opted for brushless motors due to their ability to be controlled in real-time for precise movement trajectories and to deliver high-speed strikes (Yang et al., 2020). To improve firmware, we upgraded the EPOS4 controllers to use CANBus protocol to support real-time interaction. The Beckhoff PLC controller was replaced with an STM32-based OpenCR1.0 Controller, which allows Shimon to perform music with varied dynamics and tremolo techniques. The new system also performs embedded path planning and collision detection. Collision detection was re-coded to be optimized and more reliable. Trajectory information is sent to the EPOS4 controllers in real-time via CANBus at 1Mbps. Lastly, the new system supports path planning for a variable number of arms. Using three arms instead of four, for example, can assist in case of mechanical failure, as well as create larger, more visual motions. The firmware upgrade also allows for the creation of more complex real-time gestures. The system flow chart of this system is shown in Figure 1.

Shimon’s social gesture system consists of a head and neck featuring six degrees of freedom (Hoffman and Weinberg, 2010b). The goal for Shimon’s head was to maximize expression with minimal degrees of freedom. Therefore, the six degrees of freedom are spread across only three main joints: a neck for large gestures, a head for smaller more fluent gestures, and a face for ornamental gestures. Two of the degrees of freedom are used to rotate and tilt Shimon’s neck, another two are used to tilt and rotate the head, and the last two control facial details. Originally, the facial DoFs were used to control the top and bottom of a blinking eye. Recently, since Shimon started to create lyrics and sing, the DoFs were converted to control a newly designed mouth and eyebrows. Shimon’s neck is 3 feet long to ensure visibility for large-scale gestures. The two lower DoFs (panning the whole head and tilting the neck) are intended to create large visual effects that can easily be seen by musicians and audience members. For neck pan and tilt we use harmonic FHA-c rotary motors for their precision and silence during movement. The base supports 160 degrees of motion while the neck supports 100 degrees of motion. Shimon’s neck is designed to use a non-orthogonal angle between the pan and the tilt motors, in combination with a right-angle relationship between the joints reflected in the shell. As the pan DoF rotates, the straight neck creates an illusion of a fully articulated 3-DoF joint (Hoffman and Ju, 2014). The position for a neck tilt break happens halfway along Shimon’s neck. This makes Shimon appear as if there is another moving element at the base, and makes the robot appear more animated. The head pan and tilt are attached to Shimon’s neck, and follow the same design and movement philosophy as the neck. Having two sets of parallel joints affords the robot to create seemingly fluid interactions, which can increase Shimon’s animacy and anthropomorphism (Rogel et al., 2022). As the head movements do not require high speeds, we used Dynamixel MX-28T’s for their size. Shimon’s eyebrows and mouth are driven by the same motors for social gestures. The functions of these social gestures can be used to make eye contact with musicians, bob to the rhythm, and dance based on the beat. The head serves as a social device for musicians and audiences to better connect, synchronize and engage with the robot.

## 5 Research questions

To investigate the effectiveness of Shimon’s social and ancillary gestures for human-robot musical synchronization, we pose two main research questions:

RQ1 – To what extent can a combination of ancillary and instrumental arm gestures assist time synchronization between a human and robot musician?

RQ2 – To what extent can Shimon’s social head gestures assist time synchronization between a human and robot musician?

For research question 1, we hypothesize that the use of ancillary and instrumental gestures will improve synchronicity over no gestures.

For research question 2, we hypothesize that social head gestures will improve synchronicity more than ancillary and instrumental gestures.

## 6 Methods

According to our classification as described in Section 3, we define the arm gestures as a hybrid combination of ancillary gestures (mainly achieved through the sliding gestures of the arms) and instrumental gestures (achieved through the striking gestures of the mallets). Shimon’s head conveys social gestures which in this study are mainly used for anticipation and synchronization. According to this classification, the different gestures were designed as follows.

### 6.1 Gesture design

To address the research questions, we designed a set of social, ancillary and instrumental gestures for Shimon (videos are attached as supplementary materials).

#### 6.1.1 Social gestures

Social gesture design was focused on communicating beat through head bobs as well as informing a musician on upcoming tempo changes as shown in Table 1. Shimon would move its head at 110 beats per minute (every 0.55 s), as that is the default tempo of the piece selected for the study. To operate head bobs the neck would tilt up/down by 40°, and the head would accompany the tilt by 10° 0.1 s after the neck started moving to create a follow-through effect that can help make robots appear more animate and fluid (Rogel et al., 2022). For unexpected tempo changes, informed by analysis of piano performance by humans, we identified two categories of events that can be communicated through gestures: slopes and leaps. Slopes represent gradual shifts in tempo of 20 beats per minute (BPM) within a measure. Leaps are instantaneous changes of 20 BPM. We created distinct gestures for each type of tempo variation. To represent drops and leaps, the head would immediately turn to look at the user sitting in a fixed, known position (within 0.5 s), while the neck would slowly (over the course of half a measure) pan so that Shimon is facing the piano player. Shimon would then bob its head at the new BPM. To represent slopes, we designed the neck to pan towards the user, followed by tilting up for increasing tempo, or tilting down for decreasing tempo.

**TABLE 1:** Detailed descriptions of tempo variations and gestures.

Tempo variation	Tempo definition	Gestural cue	Gesture duration
Constant tempo	The tempo maintained at 110 BPM.	Head and body move up and down to the beat (Figure 3-top)	N/A
Gradual acceleration/deceleration slopes (Accelerando/Ritardando)	The tempo gradually increases or decreases by 20 BPM within a measure	Shimon’s head and body move leftward rotation one measure before a change in tempo. Shimon then looks at the human musician and bobs head downwards (Figure 3-middle) or upwards (Figure 3-bottom) at the changing tempo pace	5.0s
Sudden acceleration/deceleration leaps (Subito Accelerando/Subito Ritardando)	The tempo suddenly increases or decreases by 20 BPM.	Shimon’s head and body move leftward one measure before the tempo change, Shimon then bobs his head to a new tempo with a smaller amplitude than the slope gesture	4.1s

#### 6.1.2 Hybrid ancillary and instrumental gestures

Hybrid gesture design utilized Shimon’s arm playing system. Here, when Shimon receives a note command, the mallet would immediately move towards the note being played (ancillary gesture) followed by striking the key with the mallet (instrumental gesture).

### 6.2 Stimuli

**FIGURE 2:**
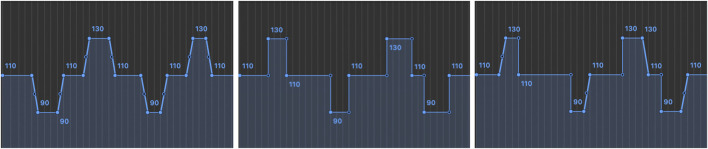
Tempo curves. Accelerando and ritardando (left). Jump and drop (middle). Combined (right).

Table 1 shows the gestures used to signify upcoming changes for slopes, leaps, and constant tempo. The gestures are pre-programmed in order to control for different subjects’ responses. We mapped different gestures to different tempo changes. Shimon performs all gestures at a constant speed, faster tempos lead to smaller head-bobbing gestures. This reflects how human ancillary and social gestures tend to decrease in size with increasing musical tempo (Wanderley et al., 2005). To evaluate the effectiveness of these gestures for communicating anticipatory timing cues to human pianists, we rearranged the piece *Pining for Spring Breeze*, composed by Yu-Hsien Teng and arranged by Stephen Hough to be played by Shimon with a variety of tempo interpretation. We decided to use a pre-composed piece for this purpose rather than one of our improvisatory systems in order to control for the gesture effect, eliminating the music played as a potential factor affecting synchronization. Four different types of tempo conditions were designed, including one rendition with constant tempo and three types of changing tempo conditions. We created 18 distinct tempo interpretations of the musical composition, with six interpretations per changing tempo condition. Figure 2 shows examples of the tempo curves associated with the three changing tempo conditions. Within these conditions, the average tempo was maintained at 110 BPM. The designed gestures and tempo curves were used as stimuli to evaluate indicative visual cues for Shimon. To study the effectiveness of communicating the tempo changes to human pianists, we measured the time deviation between the robot accompaniment notes and the correlating human pianist melodic notes. The smaller the time deviation between the human and the robot, the better their synchronization is.

**FIGURE 3:**
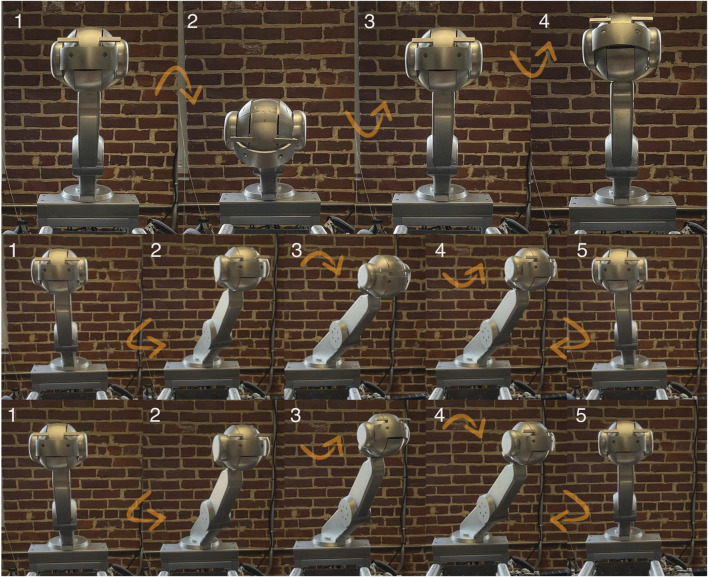
Gestures for tempo variations: Constant tempo - head bob (top); accelerating gesture (middle); decelerating gesture (bottom).

### 6.3 Participants

Thirteen pianists were recruited for the study from the Georgia Tech student body. These participants were aged 18-30, including four females and nine males, with piano-playing experience ranging from 1 to 25 years (M = 10.62, SD = 7.33). Pianists were compensated $40.00 for their time. All procedures followed the IRB Protocol H23517 issued by Georgia Institute of Technology.

### 6.4 Experiment design

The experiment was divided into two parts: objective testing and subjective testing. In the objective testing phase, participants were asked to play the melody while Shimon played the accompaniment. We conducted a 3×4 within-subjects experiment, manipulating two variables in each trial: the level of embodiment and the tempo of the music. Participants were exposed to three different levels of embodiment, including.

1. Social gestures (using head) along with ancillary-instrumental hybrid gestures (using arms)
2. Ancillary-instrumental hybrid gestures (using arms)
3. No gestures (MIDI accompaniment only for control)

Since the goal of the experiment is to study synchronization in human-robot musical performances where music-playing is essential, we included music-playing gestures in both conditions where gestures were used, and excluded a condition involving head-only gestures. To keep the three conditions as consistent as possible, we used the same MIDI file and added virtual instrument sounds through the electric piano’s speakers using a marimba sound source in Logic Pro.

For tempo, we assessed the four tempo conditions, including.

**FIGURE 4:**
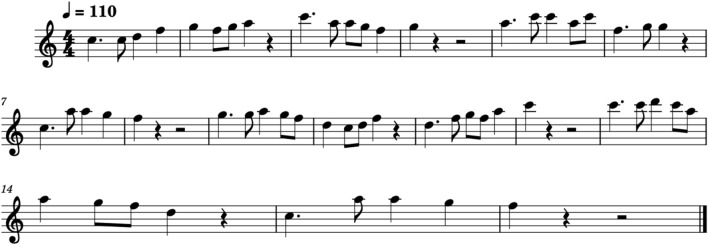
Sheet music of the piece Pining for the Spring Breeze.

1. Constant tempo
2. Gradual acceleration/deceleration slopes (Accelerando/Ritardando) shown as Figure 2 (left)
3. Sudden acceleration/deceleration leaps (Subito Accelerando/Subito Ritardando) shown as Figure 2 (middle)
4. Combined all four types of tempo changes shown as Figure 4 (right)

Each level of embodiment was evaluated for all tempo conditions. We included this level of tempo change because different types of tempo changes may have different needs for visual cues (a user can hear a gradual tempo change and slowly synchronize).

In the subjective testing phase, we designed a survey that includes the following questions.

• What is your musical background? How many years of experience do you have playing piano/keyboard?

• What are your overall thoughts about playing with a robot in this musical setting?

• How were you able to keep in time with the robot during the performance?

• Was there anything specific that the robot did that helped you maintain the timing? Please describe.

• While playing, where was your focus primarily? Was it on the music, the robot, or something else?

• Can you rate your performance in terms of staying in sync with the robot in each condition? (0 is the lowest and seven is the highest)

• What improvements or additional gestures would you suggest to enhance synchronization and the overall musical experience?

• Do you have any suggestions for improving the experience of synchronizing music performances with robots?

### 6.5 Procedure

Before the study, all participants were given the sheet music and an audio recording of the piece. We asked participants to learn the melody shown in Figure 4 before attending the study. This was to ensure that the participants would be able to focus on musicality and time synchronization, rather than getting the correct notes.

**FIGURE 5:**
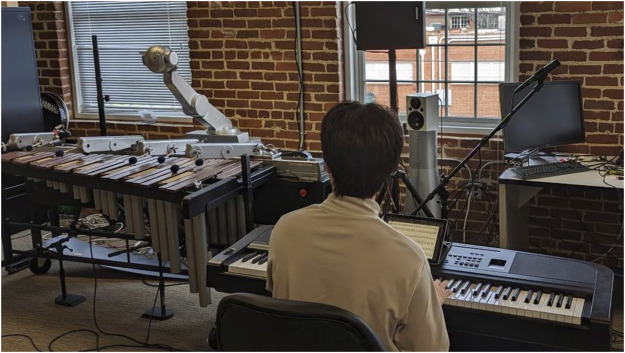
Shimon and a participant.

At the start of the study, participants entered a room with a piano facing Shimon, as depicted in Figure 5 (left). After reading and signing a consent form, they were introduced to the experimental protocols. The first part of the study functioned as a practice round to acquaint participants with a robot and re-familiarize themselves with the piece. During this session, participants performed the melody at least three times under the three levels of embodiment: no gestures from Shimon; Shimon moved arms employed arm gestures; Shimon moved both head and arms. Participants were allowed to play the piece until they could accurately synchronize with the robot without any mistakes. Only upon achieving this level of proficiency were they permitted to proceed to the following section of the study.

After the practice session, participants were instructed to synchronize their playing with Shimon’s accompaniment. Each participant played the piece under four different tempo conditions, experiencing each condition at three embodiment levels for a total of twelve trials. One of the conditions featured a constant tempo, and the other three featured tempo changes unknown to the subject throughout the piece. The order of both the tempo conditions and the embodiment levels was randomized within each session to prevent any order of exposure bias. To minimize fatigue and maintain focus, participants received a 1-min break between each session. Upon completion of the hands-on study, participants completed a subjective evaluation survey.

### 6.6 Data collection and processing

During the experiment, we recorded the MIDI notes of participants playing along with Shimon using Logic Pro. After the experiment, we compared the time deviation (the absolute value of the time difference) between the onset of each note played by the participants and the onset of each note that was supposed to be played (based on the varied tempo and sheet music) using pretty_midi library (Raffel and Ellis, 2014) in Python. This offset is known as the asynchrony value. The mean of the time deviation of all notes in each trial was then computed to represent how well the participant synchronized with the robot in that trial (known as mean absolute asynchrony).

Incorrect notes were included in the analysis because they do not influence the calculation of the time deviation; the focus remains on the timing rather than the pitch accuracy. Extra notes played by participants were excluded from the analysis to maintain consistency in the synchronization measurement. For any missing notes, the time deviation was calculated as the absolute time difference between the expected time of the missing note and the previously executed note. During data cleaning, we manually inspected each MIDI file and removed the extra notes based on our musical judgment, ensuring that only the effective notes were retained for analysis.

## 7 Results

### 7.1 Data from objective measures

To evaluate the asynchronicity between tempo conditions, we found no significant difference in between different changing tempo conditions. We therefore continued our analysis with two conditions: constant tempo and changing (all three changing tempo conditions) tempo.

**FIGURE 6:**
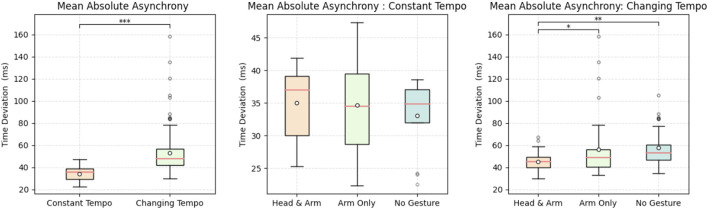
Box plot of mean absolute asynchrony under each condition.

We found that the mean absolute asynchrony in all trials within the constant tempo condition (34.25±6.52ms) were significantly lower than those in the changing tempo condition (53.09±19.75ms) . We performed a *t*-test that shows the difference was statistically significant with a $T\left(154.00\right)=-8.06,\;p<0.001^{***}$ . Figure 6 (left) displays the box plot of the mean absolute asynchrony for all trials in the constant and changing tempo conditions. To get better insights on how gestures improved each tempo condition, we analyzed each tempo condition separately.

#### 7.1.1 Constant tempo condition

Figure 6 (middle) shows the box plot of the mean absolute asynchrony for the three embodiment levels within the constant tempo condition. Specifically, the mean absolute asynchrony for the Head and Arm embodiment level was recorded at 35.00±6.01ms , for the Arm Only level at 34.66±7.90ms , and for the No Gesture level at 33.08±5.82ms . A one-way ANOVA was conducted to evaluate whether the performance time deviation across these embodiment levels among all participants was significantly different. The results indicated no significant differences [F(2,36)=0.31,p=0.7371] between the three embodiment levels in the constant tempo condition. Based on these values, we are unable to reject the null hypothesis.

### 7.1.2 Changing tempo condition

Figure 6 (right) illustrates the box plot of the mean absolute asynchrony for the three embodiment levels within the changing tempo condition. Specifically, the mean absolute asynchrony for the Head and Arm embodiment level was recorded at 45.23±8.40ms , for the Arm Only level at 56.11±27.69ms , and for the No Gesture level at 57.93±16.04ms . A one-way ANOVA was conducted to determine if the three types of gestures were significantly different from each other when the tempo was changed. The three gesture groups showed significant differences, with an F(2,114)=6.82 , $p=0.0015^{**}$ . This indicates that at least one group’s mean significantly differs from the others. The *post hoc* comparisons using Tukey’s HSD test showed that the mean difference between the Head and Arm group and the No Gesture group was significant $\left(p=0.0012^{**}\right)$ . This finding suggests that the combined use of social (head) and hybrid (arm) gestures significantly enhances human-robot musical performance synchronization. Similarly, the difference between the Head and Arm group and the Arm Only group was also significant $\left(p=0.0468^{*}\right)$ , indicating the efficacy of social gestures in improving synchronization. These results suggest that we can reject the null hypothesis. This posits that social gestures enhance performance synchronization. In contrast, the comparison between the Arm Only group and the No Gesture group did not show a significant difference (p=0.4383) . This indicates that instrumental-ancillary hybrid gestures alone do not significantly affect synchronization, thereby we cannot reject the null hypothesis.

By examining the effect of gestures in constant *versus* changing tempo conditions, we observed that gestures did not have a significant effect under the constant tempo condition. However, in the context of changing tempo, social gestures were found to enhance synchronization in human-robot musical performances.

### 7.2 Data from subjective measures

#### 7.2.1 Overall experiences

Eight participants expressed positive feelings about performing with the robot, appreciating the effort to integrate technology into artistic performance. Participants shared feedback such as, “Generally interesting, the robot head is very helpful.” Some responses highlighted that although the initial encounter with the robotic musician might evoke a sense of unfamiliarity and discomfort, the comfort level of collaboration improved over time. However, limitations were noted in the robot’s capacity for musical adaptability and emotional expressivity, underscoring the contrasts with human performers. Two participants noted that familiarity with the genre of music could enhance the overall experience. For example, “It is a very new experience for me, but it is not very enjoyable after I’m used to it, possibly because the music is not of a particular genre I like.”

#### 7.2.2 Synchronization and gestures

For the question “How are you able to keep in time with a robot?” and “Was there anything that the robot did to help?” Most participants (9 out of 13) recognized the head gestures as a significant aid in timing in changing tempo conditions, notably when the robot performed a bobbing motion or looked at the human when the tempo was changed. These gestures served as visual communication that many found crucial for maintaining the synchrony of the performance. In changing tempo conditions, eight participants also remarked on the head-turning towards them as a precursor to a tempo change, which they found to be particularly helpful for staying on beat.

#### 7.2.3 Attention focus

Participants’ focal points were categorized into two main types: some participants reported they focused their gaze on the robot if robot gestures were available, while others focused primarily on the score, keyboard, or the music itself. Specifically, Eight responses mentioned the robot, especially the gestures of its head when the head was available, as the object of their attention. One participant noted that they focused on music when there were no head gestures (in arm-only and no gesture conditions). One participant mentioned that they focused on music only in sound-only (no gesture) sessions. While the robot’s arm gestures were within the peripheral vision of the performers, they were seldom cited as a primary focus for maintaining synchronization. Five participants indicated that they focused on the audio, scorer, and keyboard. One participant reported that they focused mainly on listening to the audio but paid attention to the robot’s head gestures when it turned to indicate tempo changes in changing tempo conditions.

#### 7.2.4 Self-rating

**FIGURE 7:**
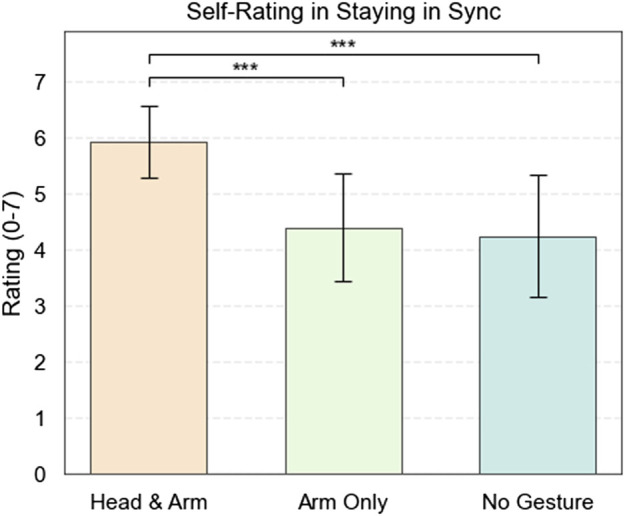
Participant self-rating in staying in sync under each condition.

Examining self-assessments of synchronization performance across different conditions indicates significant differences in participants’ average ratings. The Head and Arm condition yielded a mean rating of 5.92±0.64 , indicating markedly better synchronization compared to the Arm Only (4.38±0.96) and No Gesture (4.23±1.09) conditions as shown in Figure 7. The ANOVA results indicate a significant difference in participants’ ratings $\left[F\left(2,36\right)=13.52,p<0.001^{***}\right]$ . A Tukey HSD *post hoc* analysis shows that participants rated their performance as significantly higher in the Head and Arm condition compared to both the Arm Only $\left(p<0.001^{***}\right)$ and No Gesture conditions $\left(p<0.001^{***}\right)$ . There was no significant difference in self-rated performance between the Arm Only and No Gesture conditions (p=0.9044) . The results are consistent with the objective measurement of mean absolute asynchrony.

## 8 Discussion

**FIGURE 8:**
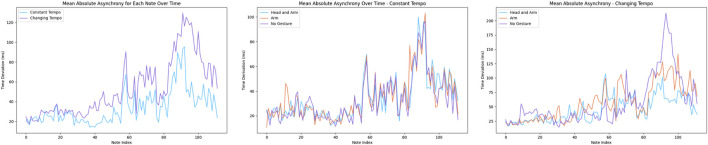
Mean absolute asynchrony of each note over time.

Through the analysis of the objective and subjective results, we found that social gestures have a significant positive effect on human-robot music synchronization, particularly in cases of changing tempo, while the impact of arm gestures alone (hybrid ancillary/instrumental gestures) is not significant. In situations where the tempo remains constant, the effects of both social and hybrid gestures are insignificant. Figure 8 shows the mean time asynchrony over time under each tempo condition. This Figure suggests that the effects of both social and hybrid gestures are most significant during more challenging segments. We also find this to be quite reasonable, as humans tend to exhibit similar behavior during performances. For instance, it has been observed that in more difficult sections of a standard concert, performers may pause, look at each other, and make larger-than-usual preparatory movements or social gestures to synchronize with other performers.

In this study, we focus on an accompaniment scenario to isolate and control for gestures alone. Specifically, we examine the role of robotic gestures in human-robot synchronization, where human musicians adapt to the robot’s pre-programmed movements, including its gestures, musical performance, and tempo variations. Previous studies have shown that mutual adaptation, based on shared information rather than one-way control, is essential for successful coordination in musical collaborations (Konvalinka et al., 2010; Lim et al., 2012; Badino et al., 2014). Our findings suggest that enhancing robot-to-human interactions through the use of social head gestures can improve human-robot synchronization in musical settings. Furthermore, integrating robots capable of both performing gestures and observing human gestures could create a system of bidirectional information flow, which would further enhance synchronization. This approach holds potential for the design of future real-time human-robot interaction systems in music performance. After establishing the role of social and ancillary gestures in an accompaniment setting, we are particularly interested in studying the role of such gestures in improvisation scenarios with Shimon such as in its previously developed ‘trading fourths’ functionality (Savery and Weinberg, 2022).

The role of performer gestures seems to stem from their biomechanical necessity. For human musicians, these gestures are crucial for preparing the body for tempo changes, as they follow a certain biomechanical structure that underlies the planning and execution of musical performance. This inherent need to physically prepare the body likely contributes to the intuitive nature of these gestures, facilitating participants’ responses to them during the study. For Shimon, the robot in our study, the mechanical needs to prepare for an acceleration or deceleration of tempo are more easily detached from each other and other gestures. This suggests that more research is needed to improve the biomechanical aspects of robotic ancillary and social gestures.

### 8.1 Limitation

Gestures in musical performance represent a complex system where any classification method may have inherent limitations, as gestures often influence one another and can, to some extent, transform from one type to another. Our classification of arm movement as a hybrid between ancillary (arm sliding) and instrumental (mallet striking) gestures does not capture the ancillary elements of striking, which could potentially help co-players predict and synchronize their gestures with the robot. While the distance between the mallet and the marimba combined with the speed of the mallet allows for only a few milliseconds of preparation for observers, we acknowledge the potential fluidity between ancillary and instrumental gestures at large. Moreover, The categorization of Shimon’s sliding gestures can fall between Instrumental and Ancillary, as they may be deemed as necessary for producing correct instrumental notes. The head gestures too could benefit from a more nuanced classification. For instance, gestures that accompany musical motion could be distinguished from those used for social interaction with fellow musicians. While we stand behind our classification and believe it can be useful for future designers and theorists, we acknowledge that more research could be conducted to better capture the complexity and multi-functionality of gestures in musical performance.

As to study limitation, during the interview portion, participants identified factors that hindered their ability to synchronize with Shimon.

1. Distracting Gestures - Shimon’s gestures were designed to grab a musician’s attention. As a result, the musician diverted too much attention away from playing the piece and towards Shimon’s head movements.
2. Too Much Tempo Variation - Tempo changes implemented during some trials did not seem musically sensible to some participants, leading to a distraction that hindered their performance of the piece. Some participants expressed their concerns regarding the lack of musicality and the unnatural feel of tempo variations:

“Most of the tempo changes did not make much musical sense to me and also happened too often, and I think that contributed to my failure to follow sometimes.”

“Maybe make the rubato feel more human with smoother transitions. Also, you could have it so certain beats can be emphasized more by the robot in addition to just slowing or speeding as that is how a real musician would likely play.”

3. Mechanical sounds of the robot - The result of the arm-only condition indicates a relatively high Interquartile Range (IQR), suggesting that the data are quite dispersed. This dispersion could be attributed to various factors, one of which may be the physical noise produced by the robot. Several participants emphasized the importance of enhancing the overall performance experience by addressing the need for mechanical design improvements aimed at reducing distracting noise from robot movement. Some participants reported:

“I think it would be fun, but the mechanisms can be a bit loud and distracting.”

“Reducing noise from robot movement would be beneficial for future improvements.”

4. Impact of Music Genre Familiarity - We only considered the participants’ piano or keyboard experience in our experiment, without accounting for their familiarity with different music genres. Two participants noted that familiarity with the genre of music could enhance the overall experience. We believe this aspect needs further research to better address subjectivity and bias.
5. Difficulty in Distinguishing Visual and Auditory Cues - The study primarily focused on the impact of visual cues (robot gestures). However, it was challenging to isolate the effects of visual cues from auditory cues (i.e., tempo changes in the music). We found that different individuals have varying primary sensory modalities when engaging in musical ensemble tasks, leading to different methods of synchronizing with the robot. Some participants primarily relied on visual cues, while others depended more on auditory cues. Although our results indicate that ancillary gestures helped participants in the human-robot synchronization task, future research could explore this issue by investigating how individuals perceive music.

Another potential limitation of our analysis includes incorrect notes in the dataset. In our data processing, we focused on synchronization accuracy, overlooking the correctness of the notes played. We believe that incorrect notes did not affect the calculation of the time difference. However, extra notes could indeed impact the results. During data cleaning, we manually removed the extra notes in each MIDI file based on our musical judgment.

## 9 Conclusion

The study investigated the influence of robot gestures on human-robot musical synchronization. The results suggest that social gestures, such as head movements, enhance synchronization performance when tempo changes are present in the music. Hybrid ancillary and instrumental (arm movements) gestures did not show a significant effect on synchronization in changing tempo conditions. Additionally, when the tempo remained constant, the influence of social, ancillary and instrumental gestures was not substantial. Furthermore, qualitative feedback highlighted the importance of reducing mechanical noise from the robot, improving the interpretation of musical tempo changes, and making robot gestures and sounds more human-like to enhance interaction and synchronization.

Overall, the study contributes to our understanding of musical gestures for both humans and robots, and the role of nonverbal gestures in human-robot musical interactions. It underscores the importance of the social and physical aspects of robotic musicians to effectively communicate and collaborate with human musicians.

## Data Availability

The raw data supporting the conclusions of this article will be made available by the authors, without undue reservation.
