# Supplementary material for: Music, body, and machine: gesture-based synchronization in human-robot musical interaction
Source: Front Robot AI. 2024 Dec 5;11:1461615. doi: 10.3389/frobt.2024.1461615 (PMC11655300; doi:10.3389/frobt.2024.1461615)

SCORE

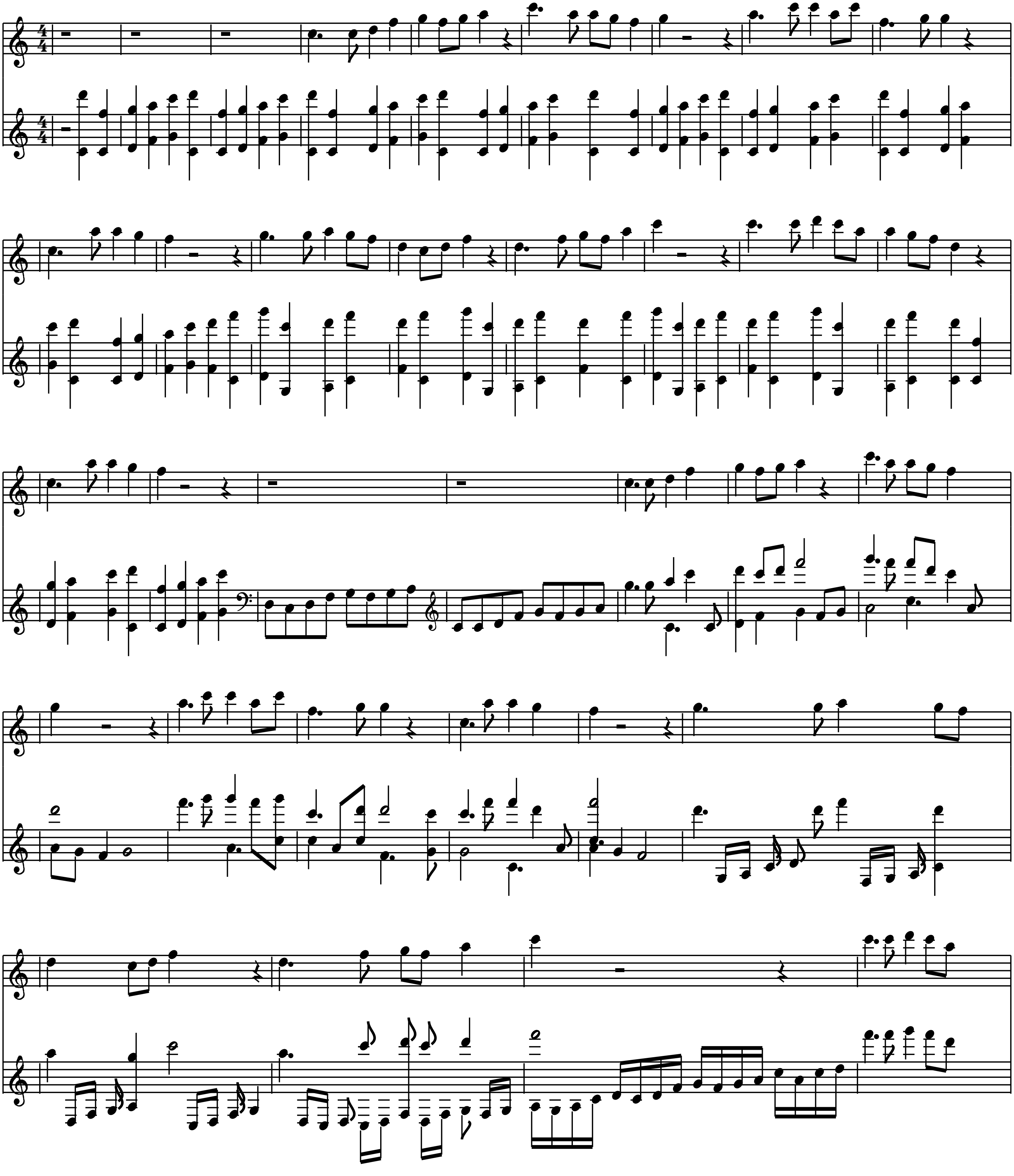

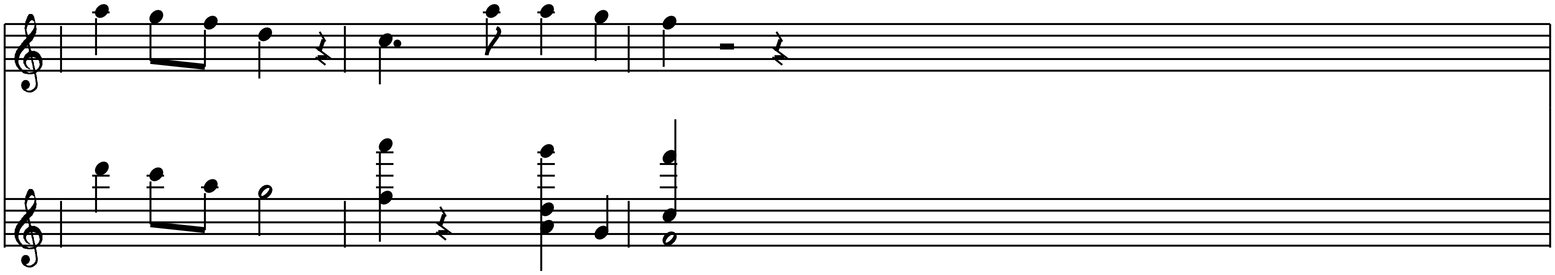

Supplement: Supplementary file 2 [file DataSheet1.pdf]
